# Screening for the C9ORF72 expansion in Greek Huntington Disease phenocopies and controls and meta-analysis of current data

**DOI:** 10.5334/tohm.61

**Published:** 2020-06-12

**Authors:** Dimitrios Rikos, Chrysoula Marogianni, Antonios Provatas, Thomas Bourinaris, Marianthi Arnaoutoglou, Pantelis Stathis, George P. Patrinos, Efthimios Dardiotis, George M. Hadjigeorgiou, Georgia Xiromerisiou

**Affiliations:** 1 University of Thessaly, University Hospital of Larissa, Neurology Department, Larissa, GR; 2 Department of Molecular Neuroscience Institute of Neurology, University College London, London, UK; 3 First Neurology Clinic, AHEPA Hospital, School of Medicine, Faculty of Health Sciences, Aristotle University of Thessaloniki, Thessaloniki, GR; 4 Department of Neurology, Mediterraneo Hospital, Athens, GR; 5 University of Patras School of Health Sciences, Department of Pharmacy, Patras, GR; 6 United Arab Emirates University, College of Medicine and Health Sciences, Department of Pathology, Al-Ain, AE; 7 United Arab Emirates University, Zayed Center of Health Sciences, Al-Ain, AE; 8 Department of Neurology, Laboratory of Neurogenetics, University of Thessaly, University Hospital of Larissa, Larissa, GR; 9 Department of Neurology, Medical School, University of Cyprus, Nicosia, CY

**Keywords:** C9orf72, meta-analysis, HD-like phenocopies, Huntington disease

## Abstract

**Background:**

Several European studies examined the role of C9orf72 repeat expansion in patients with Huntington-disease like phenotypes (HD-L). The scope of our study is to investigate the expansion frequency in a Greek HD-L cohort and the meta-analysis of all published cases. This will be of use in genetic counseling of these cases.

**Methods:**

A cohort of 74 patients with HD-L and 67 healthy controls were screened for the C9orf72 expansion status. Case-controls comparison was assessed with the Pearson’s chi-square statistic for a 2 × 2 table.

A systematic database search was conducted and seven studies, including the current study, were considered eligible for inclusion in a meta-analysis considering a total of 812 patients with HD phenocopies. Pooled mutation frequency was calculated using a Random Effects model or the Mantel-Haezsel fixed effects model, depending on the observed heterogeneity.

**Results:**

In our cohort, one patient was found to have a pathologic expansion of C9orf72, and none from the control group (chi-square: 0.91, p-value: 0.34). Pooled mutation frequency was found at 2% (CI: 1–3%) with low heterogeneity (I^2^:15%).

**Discussion:**

Based on this meta-analysis the recommendation for genetic testing for C9orf72 expansions is further solidified.

## 1. Introduction

Huntington’s disease (HD) is an autosomal dominant neurodegenerative disorder caused by an expanded CAG repeat in the huntingtin gene [1]. The CAG trinucleotide expansion above 36 repeats is pathogenic and serves as a highly sensitive and specific marker for the disease [2].

Huntington-disease like disorders (HDL) are a rare cause of chorea, resembling Huntington’s disease (HD) and its common triplet of symptoms- chorea, cognitive decline, and psychiatric symptoms, but lack the CAG expansion, responsible for the original disorder. Huntington’s disease phenocopy syndromes, represent about 1% of the cases with a clinical suspicion of HD [3]. Several neurogenetic diseases are manifested with the HD phenotype, such as “HDL1” (caused by an 8-octapeptide insertion in the *PRNP* gene), “HDL2” (caused by mutations in *JPH3*) and “HDL4” (SCA17). There is also an overlap with several spinocerebellar ataxias (SCA1, SCA2, SCA3, SCA12), dentatorubral-pallidoluysian atrophy (DRPLA) and neuroferritinopathy (caused by mutations in the ferritin light chain gene) [4]. It is crucial for clinicians to identify the genetic basis of HDL disorders, in order to organize our therapeutic strategy for the patients and provide education for their families and them. Furthermore, it can enrich the search for Huntington’s disease modifiers. In the search for possible responsible genes for HDL disorders, it was found that SCA17 accounts for 1.1%, HDL2 for 0.7%, Friedreich ataxia for 0.35% and inherited prion disease for 0.24% of HD phenocopies. Although this search is constantly expanding, most of the included diseases in the spectrum of HD like, fail to reach a robust genetic diagnosis [3].

A recent and promising scientific discovery was the identification of a mutation in the C9orf72 gene. This mutation was identified as the most common genetic cause of both familial and sporadic ALS and FTD in Caucasians [5]. In the non-coding region of C9orf72 gene, a polymorphic GGGGCC hexanucleotide repeat was found, that was detected in controls only in a few copies (less than 23 repeats), whereas in patients suffering from ALS and FTLD it was found in at least a few hundred (estimated around 700–1600) [6]. This hexanucleotide repeat was detected in other neurodegenerative diseases as well, not as strongly correlated, but with a possibly causative relation to some of them [7]. The same mutation has been studied in a large genetic screening study of HD phenocopies disorders concluding in encouraging results, relating C9orf72 and the HDL disorder [8].

In this study, we aim to investigate the frequency of C9orf72 expansions in a cohort of patients with an HD-like disorder. The included patients were tested negative for Huntington disease, and then they were screened for the GGGGCC expansion. The patients were also tested for acquired causes of chorea, but not for other genetic disorders as SCA17, PRPN mutation, FTL, ataxia-telangiectasia or DRPLA. Furthermore, we gathered all the published literature available to date relating to C9orf72 and HD-like disorders. We then performed a metanalysis of the data, to clarify the possible importance of the hexanucleotide expansion in these specific disorders.

## 2. Methods

### 2.1. Patients

A cohort of 74 patients with mainly chorea and other movement disorders (Table 1) and at least one other symptom, either cognitive decline or psychiatric symptoms, were examined by a movement disorders specialist in the outpatient clinic of AHEPA hospital and in Neurological Department of University Hospital of Larissa, during the past 10 years (2008–2018). The main criteria for the selection of these patients was the relevant clinical syndrome, that appeared to be Huntington’s disease like, after having tested negative for the Huntington chorea and other common causes of chorea i.e. infectious, immune-mediated, metabolic or structural, had been excluded either clinically or by implementing appropriate work up. One third of the patient population, apart from the compatible clinical presentation, had a relevant family history, mainly dementia-related or rarely family history of a movement disorder. A genetic test was performed for the detection of the exact number of CAG repeats, and the diagnosis of Huntington disease was excluded in all of them (<35 repeats). Patients were then classified as having a HD-like disorder. It is crucial to mention that other common and acquired causes of chorea had already been excluded, such as vascular chorea. In the study were also included healthy controls, with negative neurological history. All patients signed an informed consent form and the study was approved by the Institution’s Ethical Board.

**Table 1:** Clinical characteristics of patients characterized as HD phenocopies in the meta-analysis included.

Study	Ethnicity	Chorea n/%	Other movement disorder^1^ n/%	Cognitive impairment n/%	Psychiatric disturbance n/%	Family history n/%	Criteria for diagnosis

Hensman Moss 2014	European (not further specified)	157 (31)	57 (11.1), 84 (16.3), 42 (8.2), 73 (14.2), 35 (6.8)	176 (34.2)	60 (11.7)	105 (20.4)	Clinical diagnosis of HD
Kostic 2014	Serbian	39 (100)	31 (79.5)	29 (74)	25 (64)	?	Chorea plus two (CI, B/P, FH)
Koutsis 2015	Greek	31 (77.5)	4 (10), 8 (20), 2 (5), 6 (15), 1 (2.5)	23 (57.5)	27 (67.5)	14 (35)	1 movement disorder plus one(CI, B/P, exclusion of other causes)
Mariani 2016	Mixed	No data available					1 movement disorder plus one(CI, B/P, FH)
Baine 2018	Black-South African	No data available					Clinical diagnosis of HD
Martins 2018	Portuguese	No data available					Clinical diagnosis of HD
Current study 2019	Greek	47 (64)	12 (16), –, –, –, 15 (20)	46 (62)	53 (58)	22 (30)	One movement disorder plus one (CI, B/P), exclusion of other causes

^1^ Orderly: Dystonia; Bradykinesia; Tremor; Ataxia; Myoclonus. CI: cognitive impairment; B/P: behavioral/psychiatric disturbances; FH: family history; HD: Huntington disease.

### 2.2. Genetic analysis

DNA was extracted from all cases and controls. We screened the *C9ORF72* GGGGCC expansion in the patient cohort and the controls using the repeat-primed polymerase chain reaction (PCR) protocol as previously reported [9].

Fragment length analysis was performed on an ABI 3730XL genetic analyzer (Applied Biosystems, Inc., Foster City, CA, USA), analyzed using ABI GeneScan v 3.7 (Applied Biosystems, Inc., Foster City, CA, USA).

Confirmation of expansions and intermediate repeats was performed by Southern blotting with a 1 kb single copy probe as previously described but using BsU36I or BamHI/EcoRI restriction enzyme digests that generate a 6.2 kb or 2.4 kb band for unexpanded alleles, respectively, rather than the EcoRI digest used previously that generates an 8 kb band [10].

### 2.3. Meta-analysis

#### 2.2.1. Search strategy, study selection and data extraction

Literature databases were systematically searched using a combination of keywords “HD-like”; “Huntington disease”; “phenocopies” and “c9orf72”. The results were screened for studies investigating the relationship between HD-like cases and c9orf72 allele status. Studies with unclear case characterization and non-English language articles were excluded. The last literature review was conducted at April 10^th^, 2019. The author, year, number of genotyped cases and controls (if any), ethnicity, and c9orf72 expansion status of the participants were extracted from each study. Two independent researchers (D.R and C.M) extracted the relevant data. Any discrepancies were resolved by a third investigator (G.X). The extracted data was imported to a searchable online database.

The design of the database was described previously in detail [5]. The web database can be accessed via the following link https://neurodegenerative.herokuapp.com/. There is a publicly available version with read-only access. Administrative access can be granted to parties that are interested in adding publications.

**Figure 1:**
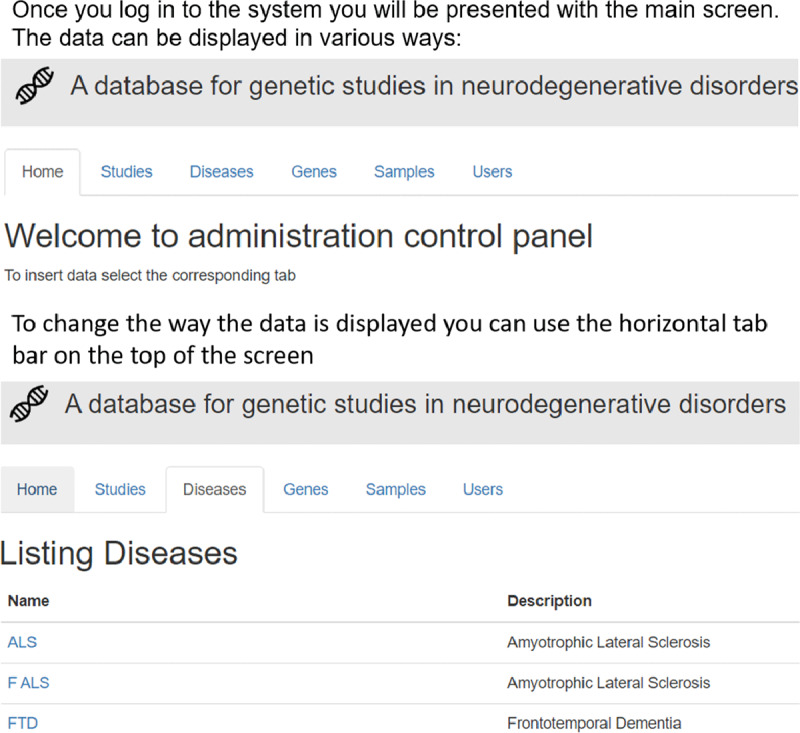
Interaction guide for the online database.

To avoid any unauthorized alteration of the data, any interested party will have to contact our system administrators to request credentials that will be specifically created for them. Usage instructions are shown graphically in Figure 1.

#### 2.3.2. Statistical analysis

Case-controls comparison of the C9orf72 status in our originally genotyped sample was assessed with the Pearson’s chi-square statistic for a 2 × 2 table. Pooled mutation frequency was calculated using a Random Effects model [11] or the Mantel-Haezsel fixed effects model [12] depending on the observed heterogeneity. Inter study heterogeneity was assessed with the I^2^ and Cochrane’s Q statistics. A Q statistic p-value lower than .10 and I^2^ greater than 50% were considered as cut-off values for statistically significant heterogeneity. Study contribution to pooled frequency and corresponding CI’s were visualized in a forest plot. Publication bias was visually assessed with funnel plots and Doi plots and numerically with the LFK index [13]. Sensitivity analysis was conducted with the leave one out method. All calculations and visualizations were conducted with the metaXL (www.epigear.com) add-in for Microsoft Excel (Microsoft Office 365 ProPlus).

## 3. Results

Demographic and clinical features of the patients with the HD-like disorders are shown in Table 1. Additionally, we screened 67 healthy controls, age and sex matched, mainly spouses. The majority of patients presented with chorea, a small number with myoclonus and a few with dystonia. Approximately 62% of them suffered from dementia, and 58% had a psychiatric disorder, mostly depression. In our cohort, one patient was found to have a pathologic expansion of C9orf72, and none from the control group (chi-square: 0.91, p-value: 0.34).

The patient with the C9orf72 mutation was a 57-year-old male patient. He presented his initial symptoms approximately 9 years ago. These symptoms were mainly choreiform movements of the extremities. The patient also suffered from depression. The clinical course of his disease gradually declined but had no signs of cognitive impairment. In fact, he had a Mini-Mental State Examination score of 29/30, while his brain MRI scan revealed generalized atrophy and white matter hyperintensities (WMH) on T2 and fluid-attenuated inverse recovery (FLAIR) MRI suggesting leukoaraiosis. From his family history, it is worth mentioning that his mother was diagnosed with cognitive decline at a relatively young age (around 55–60 years of age).

Systematic data-base search resulted in 25 studies for review. Seventeen studies were rejected as irrelevant. Another study was excluded from the analysis which assessed C9orf72 in HD-negative cases. This study failed to clarify the actual status of the patients [14]. Two studies from the same research group [15,16] potentially had overlapping cohorts and the study with the smallest sample was rejected to avoid potential bias [15]. Seven studies, including the current study, were considered eligible for inclusion in the meta-analysis [16,17,18,19,20,21] considering a total of 812 HD phenocopies (Table 2).

**Table 2:** Studies included in the meta-analysis.

Study	Cases	Positive cases	Controls	Positive controls	Ethnicity

Hensman 2014	514	10	na		Caucasian
Kostic 2014	39	1	na		Caucasian
Koutsis 2015	40	2	na		Caucasian
Mariani 2016	28	0	na		Caucasian
Baine 2018	97	0	na		South African
Martins 2018	20	1	na		Caucasian
Current study 2019	74	1	67	0	Caucasian

**Figure 2:**
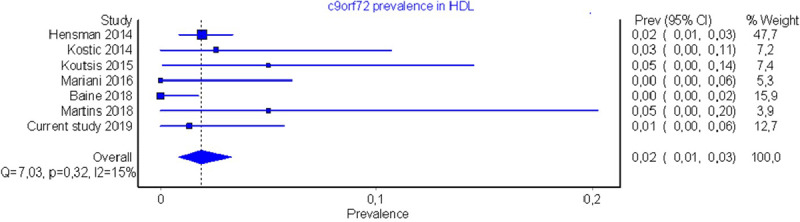
Meta-analysis Forest Plot.

**Figure 3:**
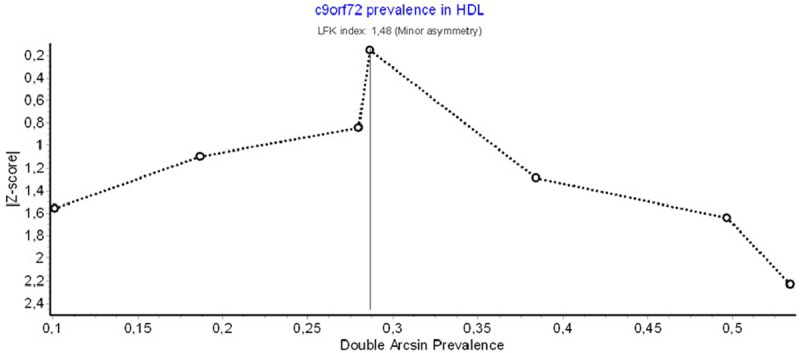
Investigation of publication bias. DOI plot.

**Figure 4:**
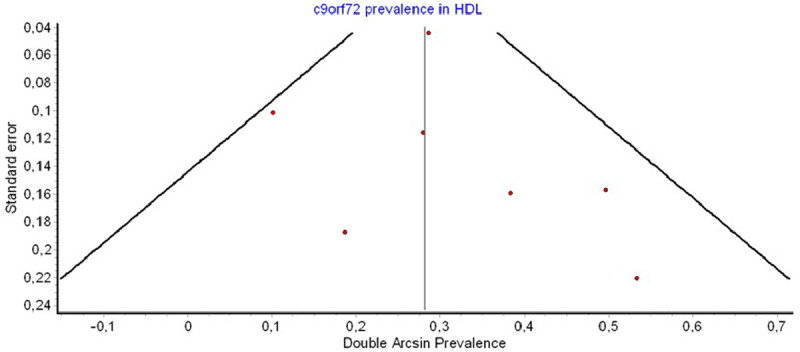
Investigation of publication bias. Funnel plot.

Pooled mutation frequency was found at 2% (CI: 1–3%) with low heterogeneity (I^2^:15%) and minor asymmetry in funnel and Doi plots (LFK index: 1.48) (Figures 2, 3, 4). Sensitivity analysis did not reveal a single study with a major effect in the overall prevalence.

Clinical characteristics of all patients included in the above studies are shown in Table 1. The total number of patients, the positive one and their ethnicity are shown in Table 2.

## 4. Discussion

HD-like phenocopies are a group of disorders that share common clinical characteristics with Huntington’s disease, but have a different genetic cause. In search of the genetic defect behind HD phenocopies, researchers found a causable mutation for at least a few of these cases. Based on the revealing results concerning ALS, FTD and C9orf72 mutation, genetic datasets of patients suffering from HD-like disorders were analyzed in search for this hexanucleotide expansion, demonstrating interesting results [22]. In particular, a study from Hensman Moss et al., showed that C9orf72 expansion is the most common genetic cause in HD phenocopies [8]. Several studies that followed verified an important relation between C9rorf72 and HD like disorders [18,19,21].

C9orf72 related disorders represent a heterogenous group of neurodegenerative diseases, as far as clinical and pathological characteristics are concerned. The pathogenic repeat expansion owes its toxic effect in the accumulation of toxic RNA foci and RNA-binding proteins, that cause secondary dysregulation of RNA splicing and trafficking. That leads to genetic instability which along with the abnormal mRNA distribution cause the formation of G-quadruplexes of multimolecular RNA. Alongside with these mechanisms, in ALS patients with c9orf72 mutation, it was found that astrocytes may transfer toxicity to motor neurons [23]. Whether the same mechanisms apply to C9orf72 positive cases of HD phenocopies remains to be elucidated. The definition of HD phenocopy syndromes seems to encompass more than just the classical triad of HD but a broader phenotype and even those patients without a clear family history of autosomal dominant neuropsychiatric disorder. In our studied cohort, the majority of patients presented with chorea as the primary movement disorder. This is significantly different compared to other studies where only 30% of the population presents chorea and the rest 70% a broader and more heterogeneous phenotype [16]. The only study that seems to follow stricter criteria for the definition of HDL is the study by Kostic at al, where all patients presented with chorea and two other inclusion criteria, either cognitive impairment, psychiatric disturbances or positive family history [18].

None of the studies used in our meta-analysis refer in detail to the currently accepted criteria for Huntington’s disease. Specifically, they do not clarify whether the patients developed motor symptoms that were “unequivocal signs of HD”, as defined in the “Diagnostic Confidence Level” (DCL) of the Unified Huntington’s Disease Rating Scale (UHDRS). According to these criteria, only subjects without genetic proof of their CAG status should be diagnosed as clinically manifest when they have a diagnostic confidence of 4. This could be a significant factor of heterogeneity of the overall studied population [24]. Comparing the characteristics of our patient with the positive patients included in the other studies in our meta-analysis, we did not find any kind of disease pattern, or at least any similarities that could be used as red flags for this particular mutation causing HD phenocopies disease.

In our study, we did not find any statistically significant result regarding C9orf72 expansion between patients and controls. Nevertheless, in the meta-analysis that followed, consisting of 7 relevant studies, including ours, the results confirmed a possible role of C9orf72 expansion in the genetic background of HD phenocopies. In the meta-analysis, the majority of the studies included Caucasian patients and only one included Afro-Americans, in which no positive patients were found. Based also in the frequency of C9orf72 expansion in ALS/FTD cohorts, an ethnicity predilection distinguishes [5]. This fact could help the clinicians prioritize the genetic tests performed to their patients. However, more studies should be conducted in Asian, Afro-American population, in order to obtain more data about these ethnicities and the frequency of C9orf72 expansion in HDL phenocopies.

The reason why there are healthy carriers of the mutation, with no clinical feature of any kind of neurodegenerative disease is not clear in the bibliography. Hypotheses exist that long term prospected studies are needed, in order to clarify that these healthy individuals with C9orf72, would not develop a relevant neurological disorder in the future. Furthermore, more data are needed about the exact number of repeat expansions that controls carry, because there is a possibility that some of them may carry a repeat number of C9orf72 around the borderline, probably justifying their healthy status. Finally, more studies should be conducted about the penetrance of the expansion and whether modified genetic factors exist, causing this kind of discrepancy.

The pathologic cutoff for C9orf72 repeat expansions remains debatable. Repeat sizes between 20 and 30 are commonly referred to as intermediate alleles and are of uncertain significance; although above 30 repeats are frequently considered pathologic. Our patient presented with approximately 200 repeats. However, the majority of the meta-analyzed studies do not refer the exact number of repeats. FTD patients with 20–22 repeats have been reported in the past without any significant clinical difference in the FTD phenotype compared with those with large expansion. Martins et al. describe 2 patients one with more than 140 repeats and the other with intermediate size of 27 repeats [21]. Kostic et al. found one patient with 270 repeats [18]. Koutsis at al did not perform Southern Blotting in order to confirm the exact number of repeats [19]. Hensman et al. found 10 cases and we confirmed the exact repeat size for 8 of them [16]. The size was between 200–250 repeats for the majority of cases. Therefore, the results do not differ from the repeats expansions seen in ALS/FTD patients or patients with other neurodegenerative disorders.

Based on this meta-analysis the recommendation for genetic testing for C9orf72 expansions is further solidified. Considering that amongst Huntington’s disease phenocopies cases only a 2.4% seems to have an apparent genetic cause according to previous studies, a pooled mutation frequency of 2% seems to have a major impact on the genetic counselling. Diagnostic test for C9orf72 is rather easy and straightforward but since this is an expansion mutation, it should be tested separately in conjunction with SCA17.

Our original study is the second study after Beck et al. [15] that investigates the C9orf72 expansion in patients and controls. All other studies examined the frequency of expansions only in patients. Pathologic expansions of C9orf72 have been occasionally detected in healthy controls. In our study, no pathologic expansions have been detected in the control group, which limits the possibility of false positives.

Our work would have been more thorough if we had included other genetic causes, like PRPN gene, FTL or SCA17 in our genetic screening. However, the main scope of our investigation is to highlight the importance of C9orf72 expansion in the investigation.
